# Biosynthesis of Cu-In-S Nanoparticles by a Yeast Isolated from Union Glacier, Antarctica: A Platform for Enhanced Quantum Dot-Sensitized Solar Cells

**DOI:** 10.3390/nano14060552

**Published:** 2024-03-21

**Authors:** Carolina Arriaza-Echanes, Jessica L. Campo-Giraldo, Felipe Valenzuela-Ibaceta, Javiera Ramos-Zúñiga, José M. Pérez-Donoso

**Affiliations:** 1 BioNanotechnology and Microbiology Laboratory, Center for Bioinformatics and Integrative Biology (CBIB), Facultad de Ciencias de la Vida, Universidad Andrés Bello, Av. República #330, Santiago 8320000, Chilejlcampo@udd.cl (J.L.C.-G.); f.valenzuelaibaceta@uandresbello.edu (F.V.-I.); j.ramoszuiga@uandresbello.edu (J.R.-Z.); 2 Doctorado en Ciencias de Materiales Avanzados, Vicerrectoría de Investigación, Universidad Mayor, Santiago 8580745, Chile

**Keywords:** *Filobasidium stepposum*, nanoparticles, QDSSC, CIS nanoparticles, Union Glacier, Antarctic yeast

## Abstract

In recent years, the utilization of extremophile microorganisms for the synthesis of metal nanoparticles, featuring enhanced properties and diverse compositions, has emerged as a sustainable strategy to generate high-quality nanomaterials with unique characteristics. Our study focuses on the biosynthesis of Cu-In-S (CIS) nanoparticles, which has garnered considerable attention in the past decade due to their low toxicity and versatile applications in biomedicine and solar cells. Despite this interest, there is a notable absence of reports on biological methods for CIS nanoparticle synthesis. In this research, three yeast species were isolated from soil samples in an extreme Antarctic environment—Union Glacier, Ellsworth Mountains. Among these isolates, *Filobasidium stepposum* demonstrated the capability to biosynthesize CIS nanoparticles when exposed to copper sulfate, indium chloride, glutathione, and cysteine. Subsequent purification and spectroscopic characterization confirmed the presence of characteristic absorbance and fluorescence peaks for CIS nanoparticles at 500 and 650 nm, respectively. Transmission electron microscopy analysis revealed the synthesis of monodisperse nanoparticles with a size range of 3–5 nm. Energy dispersive X-ray spectroscopy confirmed the composition of the nanoparticles, revealing the presence of copper, indium, and sulfur. The copper/indium ratio ranged from 0.15 to 0.27, depending on the reaction time. The biosynthesized CIS nanoparticles showed higher photostability than biomimetic nanoparticles and demonstrated successful application as photosensitizers in quantum dot-sensitized solar cells (QDSSC), achieving a conversion efficiency of up to 0.0247%. In summary, this work presents a cost-effective, straightforward, and environmentally friendly method for CIS nanoparticle synthesis. Furthermore, it constitutes the first documented instance of a biological procedure for producing these nanoparticles, opening avenues for the development of environmentally sustainable solar cells.

## 1. Introduction

Quantum dots (QDs) are semiconductor nanoparticles encompassing various compositions, including ZnS [1], CdS [2], CdTe [3], CdSe [4], PbS [5], or InP [6]. Their physical, chemical, and optical properties, including emission and absorption, can be finely tuned through the quantum confinement effect, which is dependent on size and chemical composition [7]. This versatility has led to their application in diverse fields, such as solar cell sensitization [8], organic pollutant degradation [9], hydrogen generation [10], bioimaging [3], and more.

Despite their potential, a significant challenge in the utilization of QDs lies in their associated toxicity, primarily attributed to the presence of heavy metals within the nanoparticles. These metals have been linked to the generation of reactive oxygen species (ROS) and subsequent cytotoxic effects in both eukaryotic [11] and prokaryotic [12] cells. Recent research has therefore focused on enhancing the biocompatibility of QDs to mitigate environmental impact and broaden their range of applications [13]. In this context, copper–indium–sulfide (CIS) nanoparticles have emerged as a distinctive class of QDs, characterized by low toxicity due to the absence of elements like cadmium or lead [14]. CIS QDs have garnered attention for their extensive applicability in photocatalysis [15], hydrogen production [4], biomedical applications [16,17,18], and solar cell sensitization [19,20].

Traditional chemical strategies for synthesizing CIS nanoparticles typically involve the reaction of metal precursors with 1-dodecanethiol (DDT), which serves as a sulfur source, surface ligand, and solvent [21]. While effective in producing QDs with favorable optical properties, this method presents challenges, including the necessity for high-temperature conditions, inert atmospheres, and the use of organic solvents [21,22]. Addressing these challenges, current research in the field of CIS QDs is directing its focus on developing synthesis methods with mild conditions and employing biological molecules to enhance biocompatibility. Various chemical approaches have incorporated biological molecules such as glutathione (GSH) and cysteine as reducing or capping agents [16,23,24]. In the pursuit of greener synthesis methods for CIS QDs, our group recently introduced a biomimetic approach. This one-pot method utilizes metal precursors CuSO_4_ and InCl_3_, with GSH serving as a sulfur donor and stabilizer [25]. Notably, this approach establishes a foundation for a biological method for CIS QDs synthesis, emphasizing a minimalistic yet effective approach.

Furthermore, various methods for QDs biosynthesis have been extensively documented, harnessing extremophile microorganisms to yield nanoparticles endowed with distinctive properties, such as tolerance to acidic pH [26] or NaCl [27], and viability under low-temperature conditions [28,29]. Given these strides, it is conceivable that extremophile microorganisms possess the capability to synthesize intricate nanoparticles, including CIS QDs.

Antarctica, in this context, emerges as an untapped reservoir of microorganisms boasting new and unique properties. Union Glacier, situated in the southern part of the Ellsworth Mountains, approximately 1000 km from the South Pole near the 80° S parallel [30], is particularly intriguing. This location exhibits distinctive characteristics, including the scarce presence of macroscopic life, the absence of liquid water year-round due to frigid temperatures, low humidity, and prolonged periods of light and darkness. Notably, during the summer, continuous sunlight prevails 24 h a day [30,31,32,33]. Despite these inhospitable conditions, constant UV radiation prevails, further intensified by high albedo indices resulting from the reflection of solar radiation on snow and ice [30]. In this challenging environment, various microorganisms, including bacteria, fungi, and yeasts, have been successfully isolated [34,35,36,37,38,39]. Yeasts, particularly those derived from extreme environments, have garnered special attention due to their adaptive responses to environmental stress, rendering them biotechnologically promising for the production of value-added compounds [40,41], including nanomaterials biosynthesis. While yeasts such as *Candida glabrata*, *Schizosaccharomyces pombe*, *Saccharomyces cerevisiae*, and *Rhodosporidium diobovatum* have been employed for synthesizing nanoparticles of different metals, including CdS [42,43,44,45,46], PbS [47,48,49], ZnS [50], HgS [51], SeS [52,53], and CdSe [54], the synthesis of ternary QDs or indium-containing QDs in yeast remains unreported to date.

In the present study, yeast strains were isolated from Union Glacier in Antarctica to assess their potential for synthesizing CIS QDs with unique properties, with a subsequent evaluation of their application in quantum dot-sensitized solar cells (QDSSC). To the best of our knowledge, this marks the first report on the biosynthesis of high-quality CIS nanoparticles, presenting an uncomplicated, efficient, and environmentally friendly method for producing CIS nanomaterials of substantial technological significance.

## 2. Materials and Methods

### 2.1. Chemicals

Copper sulfate (CuSO_4_), indium chloride (InCl_3_), reduced L-glutathione (GSH), titanium oxide nanoparticles (TiO_2_) with a nanoparticle size of approximately 21 nm and an anatase crystal structure, and sodium hydroxide (NaOH) were all purchased from Sigma-Aldrich (Burlington, MA, USA). Ultrapure water was purchased from Winkler (Lampa, Santiago, Chile). All chemicals were used directly without any further purification.

### 2.2. Isolation and Characterization of Yeasts from Soil Samples

#### 2.2.1. Yeast Isolation

Yeasts were isolated from soil samples collected from Ellsworth Mountains, an area near Union Glacier, Antarctica (Table 1), during the 55th Chilean Antarctic Expedition (ECA-2018) organized by the Chilean Antarctic Institute (INACH). Isolation was performed as described in Barahona et al., 2016 [34], with modifications. Antarctic samples (5 g of soil) were suspended on 5 mL YM culture medium (YM: 0.3% yeast extract, 0.3% malt extract, and 0.5% peptone, supplemented with 1% glucose and 100 µg/mL ampicillin and chloramphenicol). Tubes were pre-incubated with constant agitation at room temperature. After 12 h, aliquots of decanted culture media were seeded on 1% agar YM plates and incubated at 20 °C until colonies appeared.

#### 2.2.2. Morphology Analysis of Yeast Isolates

A drop of distilled water was deposited on top of a glass slide. A single colony was picked and distributed on top of the glass, without fixation. Then, yeast cells were observed in a MF606 microscope (BW OPTICS, Nanjing, China), equipped with a 40× objective (NA: 0.65).

#### 2.2.3. Genotypic Identification of Yeast Isolates

Genomic DNA extraction was performed as described by Barahona et al., 2016 [34]. A PCR amplification of the 5.8 rRNA/ITS region was performed using the following primers: forward (ITS1) 5′-TCCGTAGGTGAACCTGCG-3′ and reverse (ITS4) 5′-TCCTCCGCTTATTGATATGC-3′. The PCR reaction was performed using Taq polymerase (GoTaq Flexi DNA polymerase, Promega, Madison, WI, USA) according to the manufacturer’s instructions. Following an initial denaturation step at 95 °C for 5 min, the amplification reaction was carried out for 30 cycles at 95 °C for 50 s, followed by primer annealing at 55 °C for 50 s, elongation at 72 °C for 1 min, and a final extension at 72 °C for 10 min. PCR products were evaluated through 1.5% agarose gel electrophoresis and purified using the FavorPrepTM GEL/PCR Purification Kit. PCR products were sequenced in Macrogen (Seoul, Republic of Korea) using the universal primers ITS1 and ITS4. Sequences were compared with databases from NCBI.

#### 2.2.4. Growth Curves

To determine the growth rate of the yeast isolates, overnight-grown cultures of the different yeast strains were diluted 100-fold in liquid YM medium and incubated at 20 °C for 60 h with orbital agitation (160 RPM). Each hour, optical density (OD_600_) was measured using a Synergy H1 multiple-well plate reader (Biotek, Winooski, VT, USA). All growth curves were performed in triplicate.

#### 2.2.5. Copper Minimal Inhibitory Concentration (MIC) Determination

Cultures of the different yeast strains at OD_600_ ~ 0.6 were diluted 100-fold in liquid YM medium supplemented with various concentrations of CuSO_4_ ranging from 1 to 10 mM. Cultures were incubated at 20 °C for 48 h with orbital agitation (160 RPM). Growth was evaluated visually, with the lowest concentration of CuSO_4_ that inhibits the growth of strains being considered the MIC. Determinations were performed in triplicate.

#### 2.2.6. Hydrogen Sulfide (H_2_S) Production

H_2_S production was evaluated as described previously [55,56]. Yeast isolates were grown in liquid YM medium at 20 °C for 48 h. Then, cells were collected by centrifugation and washed twice with sterile distilled water. Cells were resuspended in sterile distilled water supplemented with 100 mM cysteine or 40 mM glutathione (GSH), with a value of OD_600_ ~ 0.6. A piece of filter paper soaked in a lead acetate solution (5%) was attached under the cap of the culture tubes. The tubes were incubated at 20 °C for 12 h with orbital agitation (160 RPM). Control reactions consisted of the yeast cultures without Cys or GSH. H_2_S production was detected by the change in the color of the filter paper. Pictures of all the filter papers were taken, and the H_2_S relative production was evaluated by the quantification of pixel intensity on gray scale, using the ImageJ software (http://imagej.nih.gov/ij/).

### 2.3. Biosynthesis and Characterization of CIS Nanoparticles by Yeasts Isolated from Union Glacier, Antarctica

#### 2.3.1. Biosynthesis of CIS Nanoparticles

Yeast isolates were grown in YM medium at 20 °C until reaching OD_600_ ~ 1. Cells were washed twice with sterile distilled water and then resuspended in half of the original culture volume, supplemented with CuSO_4_ (2 mM), InCl_3_ (8 mM), GSH (40 mM), and Cys (50 or 100 mM). The pH of the solution was adjusted to 9 using NaOH and the reaction was incubated at 20 °C until a change in the color of the solution indicated the presence of CIS nanoparticles. Cultures were centrifuged at 14,000× *g* for 5 min and the pellet was discarded. The supernatant containing the CIS nanoparticles was filtered with a 0.22 μm filter and concentrated in 10 kDa Amicon Tubes (Millipore, Darmstadt, Alemania). Finally, the nanoparticle solution was lyophilized using an Operon FDB-5503 lyophilizer (Yangchon-eup, Republic of Korea) 

#### 2.3.2. Absorption and Fluorescence Spectroscopy

The absorption and emission spectra of nanoparticles were recorded in a Synergy H1 multiple-well plate reader (Biotek, Winooski, VT, USA). The absorption spectrum was registered at between 300 to 700 nm. The emission spectrum was registered at between 540 to 700 nm, with a 500 nm excitation. The determination of the band-gap for the CIS nanoparticles was performed by direct transition as described in Xia et al., 2018 [57].

#### 2.3.3. Dynamic Light Scattering (DLS)

The size of nanoparticles was measured through DLS analysis on a Zetasizer Nano ZS (Malvern, UK), in distilled water.

#### 2.3.4. Transmission Electron Microscopy (TEM)

A drop of nanoparticles in suspension was left to dry out on a commercial TEM grid. TEM micrographs were obtained using a Talos F200C G2 microscope (Waltham, MA, USA) operated at 200 kV. Images were processed and analyzed using the ImageJ software (http://imagej.nih.gov/ij/). The size of the nanoparticles present in each sample was measured and plotted as frequency histograms. Additionally, the mean size of the nanoparticles was calculated.

#### 2.3.5. X-ray Diffraction Analysis (XRD)

XRD analysis was performed on the Diffractometer Bruker D8 Advance (Billerica, MA, USA) operating with Cu Kα radiation (λ = 1.5418 Å) scanning in the range 10°–80° with increments of 0.02°, as described previously [25].

#### 2.3.6. Energy-Dispersive X-ray Spectroscopy (EDX)

EDX analysis was performed using a Phenom Prox scanning electron microscope (Thermo Fisher Scientific, Waltham, MA, USA) equipped with an EDX detector.

#### 2.3.7. Photostability Assay

Biosynthesized CIS nanoparticles and biomimetic CIS nanoparticles [25] were compared for their light exposure tolerance. Both sets of nanoparticles were subjected to continuous white light exposure for 5 days, and their emission spectra were recorded following the procedure described above. Graphs were generated to analyze the percentage of fluorescence intensity, with the control condition set at 100%.

### 2.4. Application of Biosynthesized CIS Nanoparticles as Photosensitizers in Quantum Dot-Sensitized Solar Cells (QDSSCs)

QDSSCs were produced as previously described with modifications [25,58]. TEC15 fluorine-doped tin oxide coated glass (FTO glass) with a size of 20 mm × 20 mm × 2 mm, a surface resistivity of 13 [X/sq], and a transmittance of 85% was used for the construction of the cells. The cathode (counter electrode) was prepared using a solution of H_2_PtCl_6_ (50 mM) in isopropanol, deposited on the FTO by spin coating and then heated at 400 °C for 20 min. This process was repeated four times. The anode (electrode) was prepared using a solution of TiO_2_ nanoparticles, which was deposited by spin coating twice to form 2 layers of TiO_2_. The sintering was performed at 465 °C for 20 min, thus creating an active area of 1 cm^2^. The sensitization of the TiO_2_ layer was accomplished by the adsorption of CIS nanoparticles by adding 15 μL of the nanoparticle suspension on top of the TiO_2_ layer. The cell assembly was carried out by adding 14 μL of the electrolyte solution (sulfide/polysulfide NaOH 0.1 M, Na_2_S 1.0 M, and S 0.1 M in ultrapure water) to the electrode. The counter electrode was placed on it, and the cell was subsequently sealed. The cells were characterized in a solar simulator (SunLite™ Model 11002. Abet Technologies, Inc., Milford, CT, USA) under standard conditions of temperature and irradiation, with a light intensity of 100 mW cm^−2^ and AM1.5.

### 2.5. Statistical Analysis

All shown experiments were results from at least three independent assays. Using the two-way ANOVA, statistical differences were calculated. The *p*-value < 0.05 was considered significant.

## 3. Results and Discussion

### 3.1. Isolation and Characterization of Yeasts from Soil Samples from Union Glacier, Antarctica

Yeast isolation was conducted using soil samples collected from the Ellsworth Mountains, near Union Glacier, Antarctica (Table 1). Following incubation at 20 °C, three isolates, designated as A1, A2, and A3, were successfully recovered from YM plates. Initial cell morphology analysis, conducted through brightfield microscopy, revealed that all three isolates exhibited a yeast-like morphology when compared to the well-known model organism, *S. cerevisiae* (see Figure S1). To delve deeper into the taxonomic classification of these isolates, the 5.8S rRNA/ITS region was amplified and sequenced. The resulting sequences were then cross-referenced with databases hosted by the National Center for Biotechnology Information (NCBI). The identification outcomes revealed that isolates A1, A2, and A3 corresponded to *Naganishia uzbekistanensis*, *Naganishia vishniacii*, and *Filobasidium stepposum*, respectively (Table 2). Interestingly, prior research has documented the presence of the genus *Naganishia* in Antarctic territory [35,59]. Conversely, psychrophile strains of *F. stepposum* have been previously isolated from environments characterized by cold temperatures [60,61]. Remarkably, this study marks the first isolation of *F. stepposum* from Antarctic territory, contributing novel insights into the microbial diversity of this extreme environment.

A growth rate analysis at 20 °C was conducted for all three isolates to ascertain their respective growth kinetics. The time required for each yeast species to attain an OD_600_ between 0.8 and 1.0 was determined, as this range is reported as a common growth level necessary for nanoparticle biosynthesis [62]. The *N. uzbekistanensis* isolate achieved an OD600 value of 0.8 after 42 h of culture (Figure S2A). In contrast, both *N. vishniacii* and *F. stepposum* isolates reached the same OD_600_ value after 60 h of growth (Figure S2B,C).

Given the continuous exposure to CuSO_4_ required for the biosynthesis of CIS nanoparticles, the MIC value to CuSO_4_ was determined for all yeast isolates. Copper toxicity to microorganisms is known to be concentration-dependent. When copper exceeds the optimal concentration, it adversely affects several cellular targets, including membrane destabilization, ROS production, the displacement of Fe-S centers, protein damage, enzyme inhibition, and genotoxicity [63,64], while indium is considered a non-toxic metal [65]. The MIC values for *N. uzbekistanensis* (A1), *N. vishniacii* (A2), and *F. stepposum* (A3) isolates were 5.0 mM, 4.4 mM, and 3.1 mM, respectively. In comparison, the measured MIC value for *S. cerevisiae* was 2.3 mM, aligning with previous reports [66,67]. This outcome suggests that all three yeast strains from Union Glacier exhibit a higher tolerance to CuSO_4_ than *S. cerevisiae*, indicating that the exposure to CuSO_4_ during biosynthesis conditions (as described in the materials and methods) is unlikely to adversely affect yeast cells.

**Figure 1:**
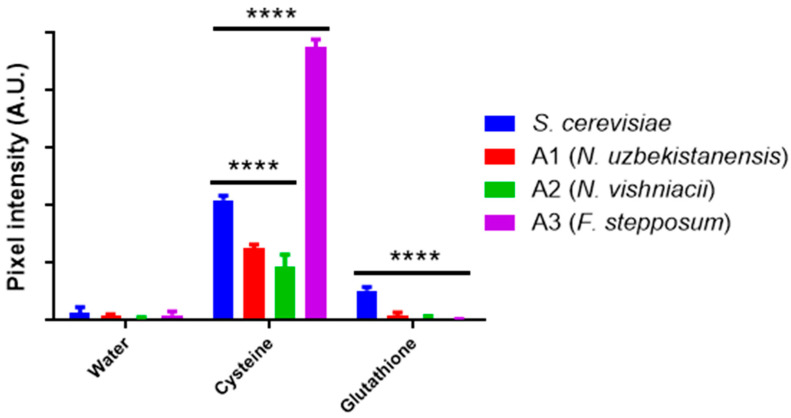
Quantification of hydrogen sulfide production. Pixel quantification associated with hydrogen sulfide production by reaction with lead acetate. Two-way ANOVA, *p*-value < 0.0001, significant difference is indicated by ****.

The significance of hydrogen sulfide (H_2_S) production in the biosynthesis of sulfur-based nanoparticles has been emphasized in previous studies [28,68,69]. To assess this aspect, the H_2_S production of all three isolates was measured using two common sulfur sources for nanoparticle biosynthesis: cysteine [27,58,70] and reduced L-glutathione (GSH) [26,71]. The presence of black spots on the filter papers in each assay (Figure S3) indicated H_2_S production by all yeast isolates in the presence of cysteine. Quantitative analysis of pixel intensity revealed that the *F. stepposum* isolate exhibited significantly higher H_2_S production compared to the other isolates (Figure 1). Conversely, H_2_S production in the presence of GSH was notably low when compared to production in the presence of cysteine (Figure 1). This observation may be attributed to the cysteine degradation pathway. Specifically, previous research has documented the degradation of cysteine by enzymes with cysteine-desulfhydrase activity, leading to the direct generation of H_2_S [58]. Additionally, the degradation of cysteine is faster than that of GSH, as the latter serves various cellular functions, including redox activity and heavy metal sequestration, among others [71]. It is crucial to note that the inability of yeast isolates to produce H_2_S when exposed to GSH does not discount the potential role of GSH in CIS nanoparticles biosynthesis. GSH has been previously recognized as a reducing agent and stabilizer in alternative methods for CIS synthesis [16,23,24,25].

### 3.2. Biosynthesis and Characterization of CIS Nanoparticles by Yeasts Isolates

The biosynthetic capacity of all yeast isolates to produce CIS nanoparticles was assessed using precursor concentrations established in our previously defined biomimetic synthesis method [25]. The biosynthesis reaction utilized CuSO_4_, InCl_3_, GSH, and, based on the observed H_2_S production (Figure 1), cysteine as a sulfur source. Five distinct biosynthesis conditions were examined, varying in the presence of GSH or cysteine, as well as the concentration of cysteine. The progress of the biosynthesis reaction was monitored daily, and color changes in the solution were observed, transitioning from yellow to orange and eventually red. These color changes correspond to the spectroscopic characteristics of CIS nanoparticles, where both absorption and emission spectra fall within the visible light spectrum [72]. Figure S4 displays reaction tubes after 14 days of incubation. In the control reaction, with yeast cells resuspended in water, no discernible color change was noted (Figure S4), suggesting that alterations in solution color are attributed to the addition of biosynthesis precursors rather than the generation of colored compounds or pigments produced by yeast isolates. Furthermore, reactions conducted without yeast cells failed to produce the expected color indicative of CIS nanoparticle synthesis, emphasizing the indispensability of yeast cells for this process. For *N. uzbekistanensis* and *N. vishniacii* isolates, only a subtle color change was detectable after the various biosynthesis conditions, possibly indicating a longer incubation period or the necessity for different sulfur sources for effective CIS nanoparticle biosynthesis, consistent with reports in other microorganisms [73]. Conversely, the *F. stepposum* isolate exhibited noticeable color changes associated with CIS nanoparticle synthesis when the reaction was performed with GSH or with the addition of two different concentrations of cysteine (Figure S4, conditions 1–3). Intriguingly, no color change occurred when GSH was absent from the reaction (Figure S4, conditions 4 and 5). This strongly suggests the pivotal role of GSH in the formation and stability of biosynthesized CIS nanoparticles. Similar roles for GSH have been reported in the biosynthesis of other QDs [26,71], as well as biomimetic QDs [25,74].

**Figure 2:**
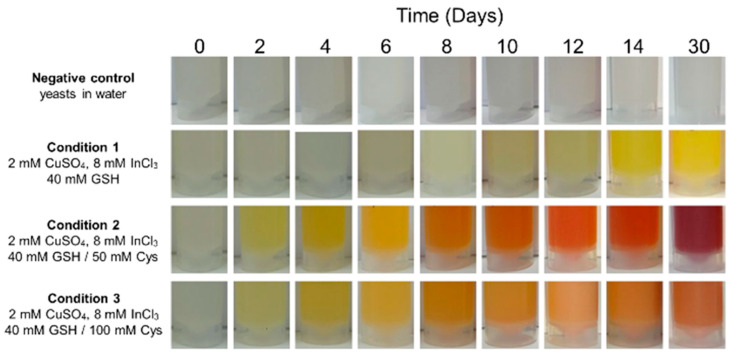
CIS nanoparticles kinetics production by *F. stepposum*. Photographs of tubes containing yeast cells of *F. stepposum* under indicated biosynthesis conditions at different incubation times at 20 °C. Biosynthesis was conducted using 2 mM CuSO_4_ and 8 mM InCl_3_. Variations under different conditions were carried out using different concentrations of GSH and Cys. Negative control corresponds to yeast in water. Condition 1: 40 mM GSH; Condition 2: 40 mM GSH and 50 mM Cys; Condition 3: 40 mM GSH and 100 mM Cys.

Time-dependent variations in the color of the reaction were assessed using the *F. stepposum* isolate for CIS biosynthesis under previously tested conditions (Figure S4, conditions 1–3). When the reaction involved CuSO_4_, InCl_3_, and GSH, a yellow color emerged in the solution starting from the 10th day of incubation, intensifying over time until 30 days were reached (Figure 2, condition 1). In the presence of 50 mM of cysteine, the same yellow color appeared from the second day of incubation (Figure 2, condition 2). This color transitioned over time, displaying an orange hue starting from the 8th day of incubation and turning red by day 30. Under conditions with 100 mM of cysteine (Figure 2, condition 3), the solution also changed to yellow from day 2 of incubation and transitioned to orange on day 8. However, the solution retained this color even after 30 days of incubation. These findings suggest that the addition of cysteine enhances the biosynthesis of CIS nanoparticles by the *F. stepposum* isolate, likely mediated by the release of H_2_S from the cells. Nanoparticles synthesized at 14 and 30 days under conditions 1, 2, and 3 (Figure 2) were purified (as described in materials and methods) and subjected to further characterization.

**Figure 3:**
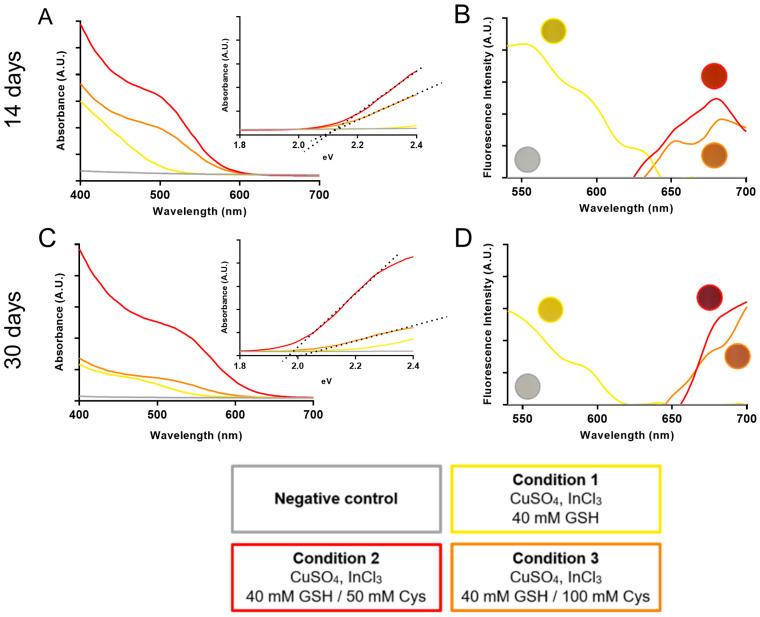
Optical properties of biosynthesized CIS nanoparticles. Absorbance and fluorescence emission spectrum for CIS nanoparticles biosynthesized at different conditions. (**A**,**B**) A total of 14 days biosynthesis, and (**C**,**D**) a total of 30 days of biosynthesis. (**A**,**C**) include band-gap calculation (inset).

The spectroscopic properties of biosynthesized nanoparticles were initially assessed. The absorbance spectra of nanoparticles synthesized after 14 days, with 50 or 100 mM of cysteine, exhibited a peak near 500 nm, indicating the characteristic absorption of CIS nanoparticles (Figure 3A). The band-gap value calculated from these spectra was 2.1 eV (Figure 3A, inset). Notably, when the synthesis was conducted without cysteine, no discernible peak was observed. In terms of the emission spectra, nanoparticles synthesized in the presence of cysteine displayed a peak between 650 and 700 nm (Figure 3B). In contrast, nanoparticles synthesized without cysteine did not exhibit the expected peak associated with CIS nanoparticles. Comparatively, the absorbance spectra of nanoparticles synthesized after 30 days, with 50 mM of cysteine, showed a peak shift to 550 nm (Figure 3C) compared to the 14-day reaction (Figure 3A) and presented a band-gap value of 1.95 eV (Figure 3C, inset). In reactions conducted without cysteine or with 100 mM cysteine, the absorbance spectra displayed a decrease in intensity, with a less defined peak (Figure 3C). The emission spectra of nanoparticles synthesized at 30 days exhibited similar behavior compared to those at 14 days. However, synthesis reactions performed in the presence of cysteine showed a shift beyond 700 nm. As previously reported, the change in solution color is a characteristic feature of CIS nanoparticles, and as observed here, the absorbance and emission spectra evolve over time [25]. These findings affirm that the addition of cysteine enables the biosynthesis of CIS nanoparticles, and that varying the cysteine concentration and incubation time provides control over the optical properties of the resulting nanoparticles.

The reactions conducted with 50 and 100 mM cysteine at both 14 and 30 days exhibited distinctive absorbance and emission spectra characteristic of CIS nanoparticles [25], prompting the further analysis of size and morphology. For hydrodynamic size measurement, the purified nanoparticles were subjected to DLS study. The analysis of nanoparticles synthesized after 14 days with 50 mM cysteine revealed a population with sizes ranging from 2 to 10 nm, a mean size of 4.45 nm, and a polydispersity index (PdI) of 0.233 (Figure S5A). In the 30-day reaction, nanoparticles displayed a size range of 3 to 15 nm, a mean size of 8.84 nm, and a PdI of 0.311 (Figure S5B). The reaction with 100 mM cysteine, at both 14 and 30 days, generated nanoparticles size between 5 and 20 nm, with mean sizes of 14.54 nm (PdI 0.521) and 14.82 nm (PdI 0.531), respectively (Figure S5C,D). This indicates that increased incubation time, in the presence of 50 or 100 mM cysteine, leads to an increase in nanoparticle size, correlating with the observed shifts in absorbance and emission spectra (Figure 3).

**Figure 4:**
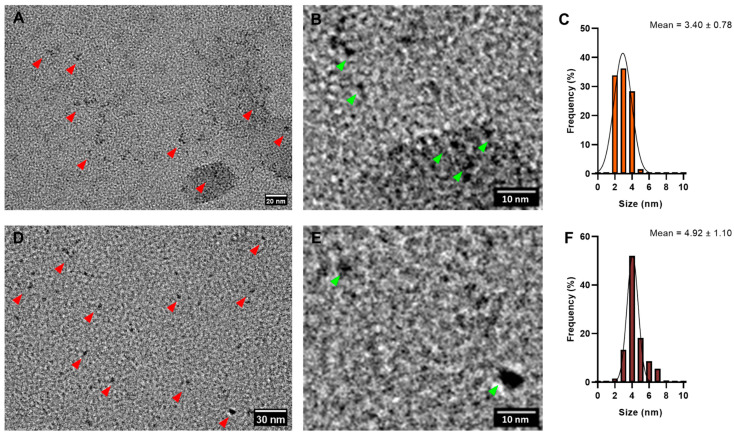
Size characterization of biosynthesized CIS nanoparticles. TEM micrographs of nanoparticles synthesized with 50 mM cysteine. (**A**) Nanoparticles of 14 days of biosynthesis, marked by red arrows. (**B**) Digital zoom of (**A**), highlighting pyramidal nanoparticles by green arrows. (**C**) Size histogram of nanoparticles from (**A**). (**D**) Nanoparticles of 30 days of biosynthesis, marked by red arrows. (**E**) Digital zoom of (**D**), highlighting pyramidal nanoparticles by green arrows. (**F**) Size histogram of nanoparticles from (**D**).

As an additional method for morphology analysis, nanoparticles synthesized with 50 mM cysteine at 14 and 30 days were examined using TEM. Despite spectroscopic and size characteristics aligning with CIS nanoparticles biosynthesized by the biomimetic method [25], TEM micrographs revealed an heterogeneous nanoparticle population (Figure 4A,D), with some nanoparticles displaying triangular-shaped morphology (Figure 4B,E). While most reported CIS nanoparticles are spherical [4,21,25], there are instances of pyramidal-shaped nanoparticles as well [14,75,76]. Nanoparticles synthesized at 14 days exhibited a mean size of 3.4 nm (Figure 4C), while those synthesized at 30 days had a mean size of 4.9 nm (Figure 4F). Consistent with the hydrodynamic size measured by DLS, the nanoparticle size increased with prolonged incubation time. Notably, the biosynthesized nanoparticles were relatively small compared to biomimetic CIS nanoparticles (mean size of 33.5 nm) [25]. However, various synthesis methods for CIS nanoparticles can yield crystals within a wide size range, from 2 nm to 300 nm [77,78], placing the size of biosynthesized nanoparticles within the expected range. Another noteworthy observation is the disparity between the hydrodynamic size measured by DLS and the size measured by TEM. This phenomenon is consistent with findings in other studies involving biological nanoparticles. The variation could be attributed to the diverse forms visualized by TEM (Figure 4) or the biological coating characteristic of biological nanoparticles, both of which influence the hydrodynamic size [79,80].

To further analyze the structure of the nanoparticles, XRD analysis was performed. The previous XRD analysis of biomimetic nanoparticles revealed a tetragonal crystalline structure, characterized by three main peaks at 27.93, 46.55, and 55.01° [25]. The obtained diffractograms for both nanoparticles, biosynthesized at 14 and 30 days, exhibit a predominant amorphous component alongside a secondary crystalline component (Figure S6). Although peaks of the diffractogram could suggest that the crystalline component corresponds to chalcopyrite CIS nanoparticles [81], because of the area of the amorphous component (marked by a red line in Figure S6), it is difficult to find well-defined peaks that could back this evidence. It is documented that nanoparticles prepared by biological methods or functionalized with organic components, such as proteins, often exhibit an amorphous structure [82]. This finding is consistent with our previous TEM and DLS analyses, further indicating that biosynthesized nanoparticles possess an organic coating that may influence their properties.

**Figure 5:**
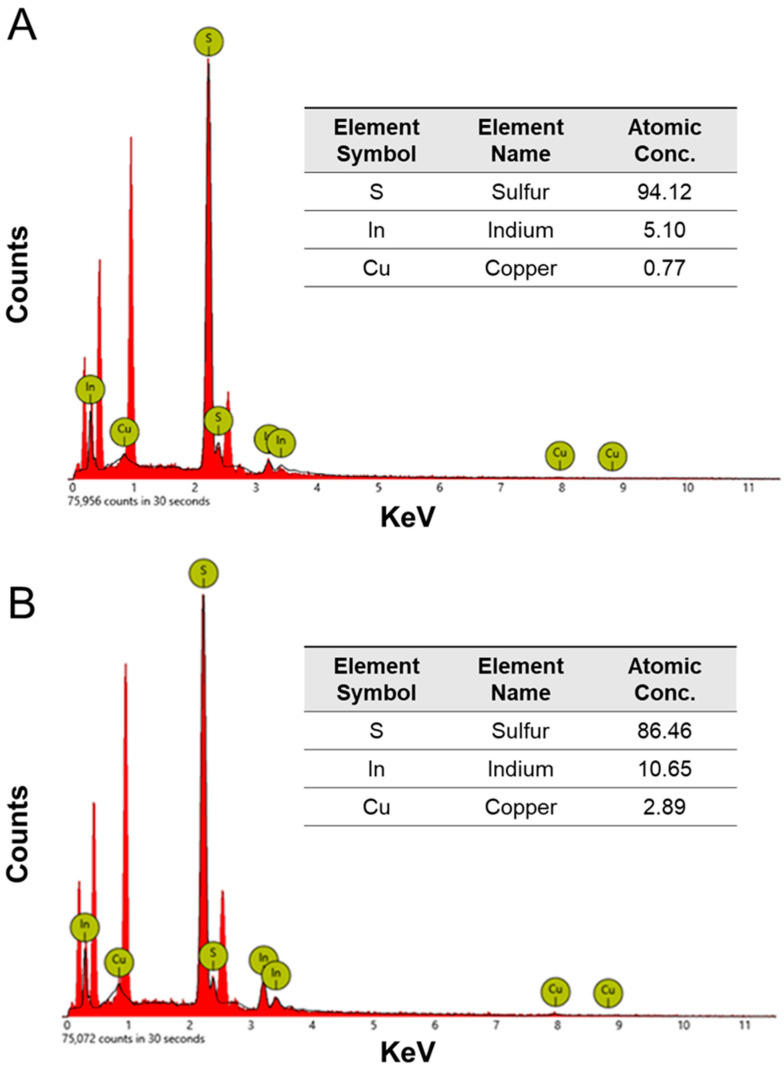
Evaluation of the composition of the biosynthesized nanoparticles. Energy-dispersive X-ray spectroscopy (EDX) analysis: (**A**,**B**) correspond to the syntheses performed with 50 mM cysteine at 14 and 30 days, respectively.

CIS nanoparticles synthesized under condition 2 (50 mM cysteine) at different incubation times (14 and 30 days) underwent EDX analysis (Figure 5). The presence of copper, indium, and sulfur was confirmed, with varying copper/indium ratios determined for each incubation time (0.15 for the 14-day synthesis and 0.27 for the 30-day synthesis). The high sulfur content in the nanoparticles may result from thiol capping provided by both cysteine and GSH used in the biosynthesis, forming part of the organic matter coating inherent to biological nanoparticles [71]. This result indicates an increase in the amount of copper with extended incubation time and that the produced CIS nanoparticles exhibit a non-stoichiometric composition, deviating from the usual 1:1:2 (Cu:In:S) proportions [83,84].

In summary, our characterization experiments lead us to conclude that, under biosynthesis conditions, the isolated *F. stepposum* strain synthesizes amorphous CIS nanoparticles capped with GSH. These nanoparticles, depending on the reaction time, exhibit a size ranging from 3.4 to 4.92 nm and a copper/indium ratio ranging from 0.15 to 0.27.

**Figure 6:**
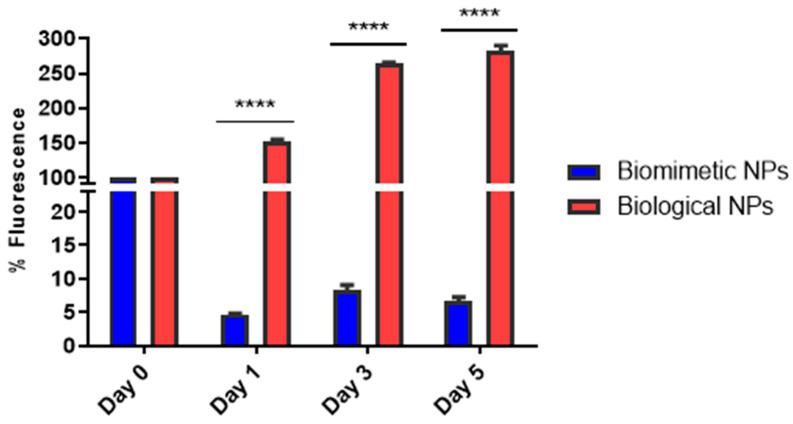
CIS nanoparticles photostability evaluation. Nanoparticles were exposed to continuous light for 5 days and fluorescence emission was measured on different days (0, 1, 3, and 5 days). Two-way ANOVA, *p*-value < 0.0001, significant difference is indicated by ****.

Given that the *F. stepposum* species was isolated from Union Glacier with continuous exposure to UV light [30], we aimed to explore the photostability of the synthesized nanoparticles. Photostability assays were conducted with CIS biosynthesized nanoparticles incubated for 30 days (condition 2, 50 mM cysteine), given that their spectroscopic characteristics are more closely related to CIS nanoparticles synthesized through the biomimetic method [25]. The photostability of CIS biosynthesized nanoparticles was compared to that of CIS nanoparticles synthesized through the biomimetic method, both subjected to continuous exposure to white light over a 5-day period (Figure 6). Biomimetically synthesized nanoparticles (depicted by blue bars) exhibited a decrease of over 90% in their fluorescence emission after 24 h of light exposure. In contrast, biosynthesized CIS nanoparticles (illustrated by red bars) demonstrated greater stability to light exposure throughout the entire assay and, notably, increased their fluorescence emission with the incubation days. This phenomenon aligns with the concept of photoactivation or photoenhancement observed in other semiconductor nanoparticles like ZnSe and PbS types [85,86,87]. The photoactivation of nanoparticles in aqueous solutions induced by light exposure has been attributed to a process of photocorrosion. During this process, photoinduced charge carriers located at the surface of the nanoparticle interact with oxygen in the solvent. This interaction gradually erodes the atomic-scale topographic flaws on the nanoparticle’s surface, resulting in smoother crystals with enhanced fluorescence emission [88]. While mentions of photoactivation with CIS QDs have been reported in the context of photoelectrochemical studies [20,89], there is a lack of studies exploring photoactivation induced by light exposure on this type of nanoparticle.

### 3.3. Application of Biosynthesized CIS Nanoparticles as Photosensitizers in QDSSCs

One of the primary applications of CIS QDs lies in their utilization as photosensitizers in solar cells. Considering the high photostability of the biosynthesized nanoparticles (Figure 6), the effectiveness of biosynthesized CIS QDs as photosensitizers was assessed in comparison to CIS QDs synthesized through the biomimetic method [25]. The solar cell parameters are detailed in Table 3 and the current–voltage curves are shown in Figure S7, encompassing an efficiency comparison between solar cells featuring a TiO_2_ layer and those photosensitized with either biological CIS or biomimetic CIS. Cells incorporating the addition of QDs exhibited higher efficiency values than those with only TiO_2_. However, upon scrutinizing the cells photosensitized with CIS QDs, it was observed that biosynthesized CIS yielded lower efficiency compared to the biomimetic CIS employed in this study (Table 3).

The efficiency values obtained were juxtaposed with solar cells employing various photosensitizers, as outlined in Table 4. Notably, the efficiency of biosynthesized CIS surpassed that of QDSSCs photosensitized with biosynthesized CdS and Ag_2_S QDs, which exhibited efficiencies of 0.0016% and 0.0054%, respectively [58,90]. Moreover, in comparison with ternary QDs like CdSAg (0.0222%), biosynthesized CIS demonstrated comparable efficiencies, underscoring the significance of the QD type synthesized for use as a photosensitizer.

The existing literature on QDSSCs utilizing biosynthesized QDs primarily focuses on biosynthesis in plants and the utilization of TiO_2_ and ZnO NPs as photoanodes [93]. To broaden the comparison, we also assessed the efficiency values obtained with other types of cells that can accommodate biological photosensitizers, such as dye-sensitized solar cells (DSSCs). DSSCs incorporating pigments obtained from Antarctic bacteria demonstrated efficiencies of 0.008% [91] and 0.0332% [92]. When contrasting these efficiency values with those obtained from biosynthesized CIS QDs, the results suggest similar efficiencies, possibly indicating a slightly lower efficiency due to the organic nature of the CIS QDs.

In Figure S8, a schematic depicts the operational mechanism of QDSSC. It illustrates the electron transfer from the conduction band of the CIS nanoparticle to the conduction band of the TiO_2_ nanoparticles, positioned at a lower energy level. Subsequently, this transferred electron is harnessed through the circuit for energy generation [94,95]. However, it is worth noting that biosynthesized nanoparticles include a layer of organic matter, which could pose challenges to the electron transfer process. Despite these challenges in enhancing efficiency, factors such as the cost and stability of photosensitizers used in solar cells [96] are crucial considerations. Therefore, research on photosensitizers of QDs obtained through biological synthesis is gaining traction in these areas of competition.

## 4. Conclusions

In this study, we assessed various conditions for obtaining CIS QDs through biosynthesis. We successfully reported the biosynthesis of CIS QDs using a simple method with yeast isolated from a soil sample of Union Glacier in Antarctica. Nanostructures generated from biosynthesis exhibited spectroscopic characteristics of CIS QDs, featuring triangular morphology with a size ranging between 3 and 5 nm, regulated by the incubation time. Finally, we evaluated the performance of the biosynthesized CIS QDs through their photostability and application in solar cells, achieving higher photostability than their chemically synthesized counterparts. This also demonstrated their potential as photosensitizers in solar cells. In summary, the obtained results represent the documentation of *F. stepposum* yeast present in Antarctic soil and its utilization for the biosynthesis of CIS nanoparticles. Although there is still room for optimization studies for potential applications and the evaluation of biosynthesis with other yeasts or microorganisms, this work stands as the first report on the biosynthesis of high-quality CIS nanoparticles by yeast isolated from Antarctic soil.

## Figures and Tables

**Table 1:** Soil samples obtained from Union Glacier in Antarctica.

Sample Code	Coordinates
GUMRC-5B-2018	79°47′26.1″ S/82°55′43.2″ E
GUNRC-1C-2018	79°47′32.5″ S/32°58′01.4″ E
GUNRC-4C-2018	79°47′27.9″ S/32°55′47.6″ E
GUNEH-1B-2018	79°47′18.7″ S/83°19′52.5″ E
GUNEH-3A-2018	79°47′14.2″ S/83°19′9.4″ E
GUNEH-1A-2018	79°47′18.1″ S/83°19′52.5″ E
GUNEH-4A-2018	79°47′21.9″ S/83°19′58.1″ E

**Table 2:** ITS sequencing analysis of isolated Antarctic yeast.

Code	Primer	Name	Query Coverage	Identity
A1	ITS1	*Naganishia uzbekistanensis*	94%	99.81%
	ITS4	*Naganishia uzbekistanensis*	100%	100%
A2	ITS1	*Naganishia vishniacii*	100%	99.81%
	ITS4	*Naganishia vishniacii*	100%	100%
A3	ITS1	*Filobasidium stepposum*	100%	100%
	ITS4	*Filobasidium stepposum*	100%	100%

Identifications were made by sequence comparison with the BLASTn match with the NCBI GenBank database.

**Table 3:** Photovoltaic parameters of QDSSC solar cells.

Cell	Open Circuit Voltage Voc (V)	Short Circuit Current Isc (A)	Pmax (W)	Fill Factor FF	Efficiency η (%)
TiO_2_	0.150	1.030 × 10^−5^	1.803 × 10^−6^	0.955	0.0017
CIS (biosynthesis)	0.247	2.729 × 10^−4^	2.247 × 10^−5^	0.367	0.0247
CIS (biomimetic)	0.335	8.951 × 10^−4^	1.047 × 10^−4^	0.349	0.1047

**Table 4:** Comparison of the efficiency of solar cells with different photosensitizers.

Photosensitizer	Efficiency (%)	References
CIS (biosynthesis)	0.0247	This work
CIS (biomimetic)	0.1047	This work
Ag_2_S QDs biosynthesis	0.0054	[58]
CdS QDs biosynthesis	0.0016	[90]
CdSAg QDs biosynthesis	0.0222	[58]
Biological pigments	0.0080	[91]
Biological pigments	0.0332	[92]

## Data Availability

The datasets used and/or analyzed during the current study are available from the corresponding author on reasonable request.

## Supplementary Materials

The following supporting information can be downloaded at: https://www.mdpi.com/article/10.3390/nano14060552/s1. Figure S1: Morphological analysis of isolated yeasts; Figure S2: Growth curves of isolated yeasts from Union Glacier; Figure S3: Hydrogen sulfide production assay; Figure S4: Evaluation of Cu-In-S biosynthesis conditions; Figure S5: Size evaluation of biosynthesized nanoparticles; Figure S6: XRD analysis of CIS nanoparticles; Figure S7: Current–voltage (I–V) curves of solar cells; Figure S8: Schematization of the operation mechanism of quantum dot-sensitized solar cells (QDSSC).
