# Supplementary material for: Biosynthesis of Cu-In-S Nanoparticles by a Yeast Isolated from Union Glacier, Antarctica: A Platform for Enhanced Quantum Dot-Sensitized Solar Cells
Source: Nanomaterials (Basel). 2024 Mar 21;14(6):552. doi: 10.3390/nano14060552 (PMC10975457; doi:10.3390/nano14060552)
Supplement: Supplementary file 1 [file nanomaterials-14-00552-s001.zip › nanomaterials-2899388-supplementary.pdf]

Electronic supplementary information (ESI)

Biosynthesis of Cu-In-S Nanoparticles by a Yeast Isolated from Union Glacier, Antarctica: A platform for Enhanced Quantum Dot-Sensitized Solar Cells

Carolina Arriaza-Echanes[1], Jessica L. Campo-Giraldo[1], Felipe Valenzuela-Ibaceta[1], Javiera Ramos-Zúñiga[1], José M. Pérez-Donoso[1*]

[1]Universidad Andrés Bello, BioNanotechnology and Microbiology Laboratory, Center for Bioinformatics and Integrative Biology (CBIB), Facultad de Ciencias de la Vida, Av. República # 330, Santiago, Chile.

*Corresponding author. Email: jose.perez@unab.cl

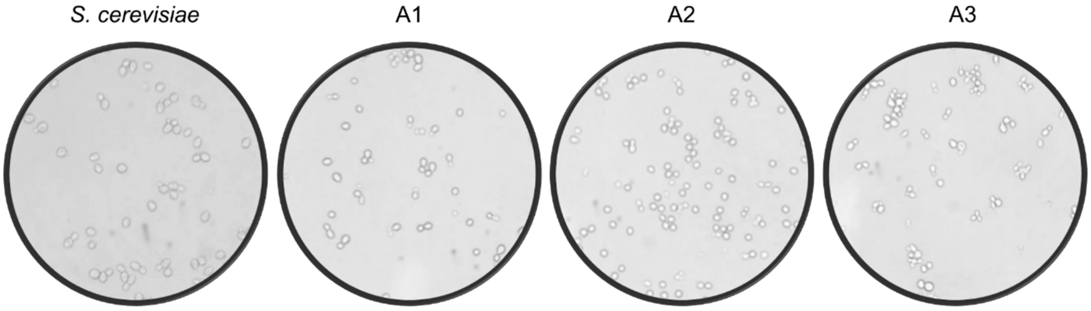

Figure S1: Morphological analysis of isolated yeasts. Representative microscopy images of isolates A1, A2, A3 and *S. cerevisiae*, taken using brightfield microscopy at 40 X magnification.

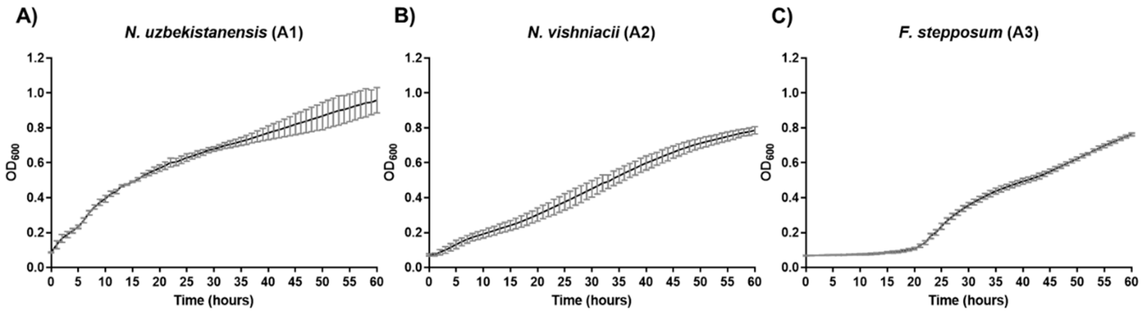

Figure S2: Growth curves of isolated yeasts from Union Glacier. (A) Growth curve of A1 isolate, *N. uzbekistanensis*. (B) Growth curve of A2 isolate, *N. vishniacii.* (C) Growth curve of A3 isolate, *F. stepposum*. All isolates were grown at 20 °C during 60 hours, with orbital agitation, and the OD600 value of cultures was measured each hour.

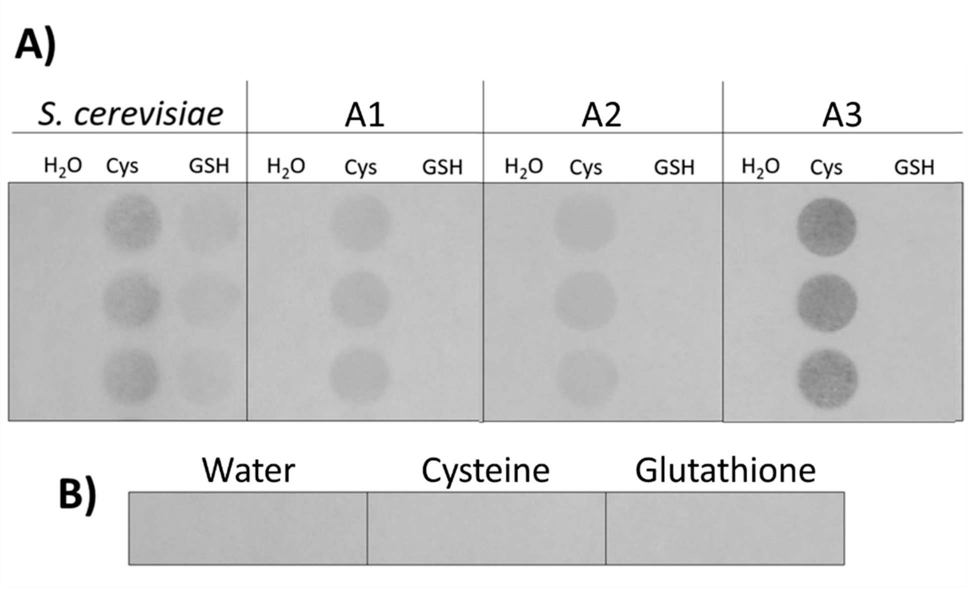

Figure S3: Hydrogen sulfide production assay. A) Filter papers showing the production of black precipitate as a consequence of sulfide interaction with lead acetate. All assays were performed with yeasts resuspended in distilled water at an OD_{600} of 0.6. H_2O: yeast in water. Cys: yeast with the addition of 100 mM cysteine. GSH: yeast with the addition of 40 mM glutathione. B) Controls without yeast.

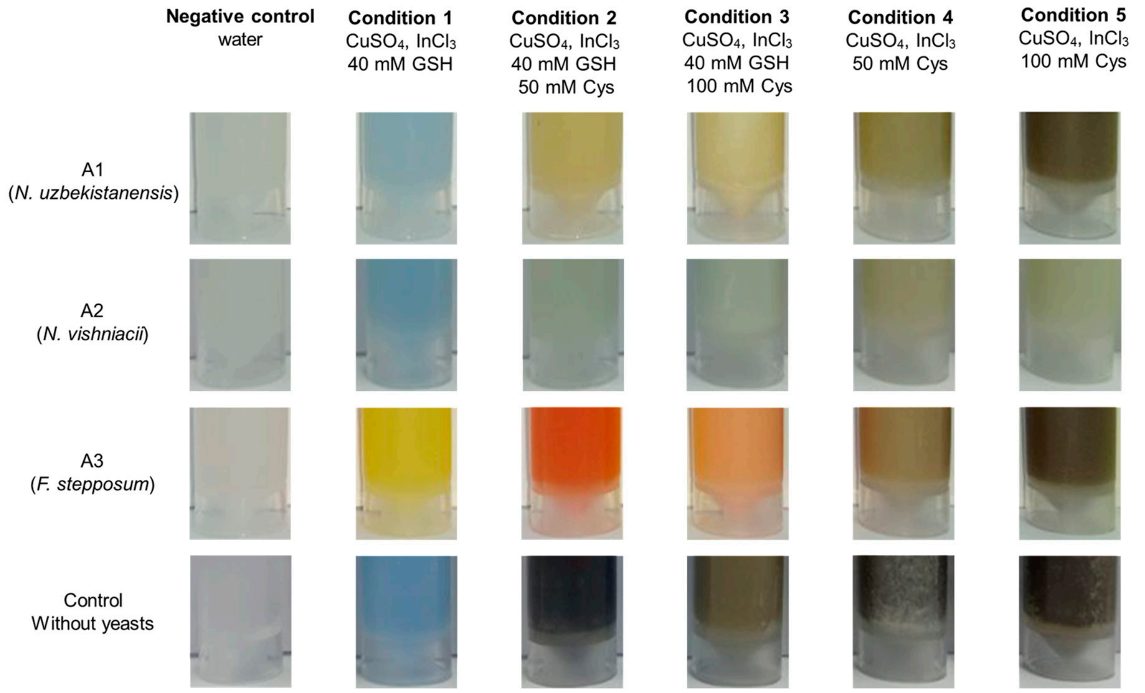

Figure S4: Evaluation of Cu-In-S biosynthesis conditions. Assays for all yeast isolates and yeast-free control conditions were performed at 20 °C and 14 days of incubation.

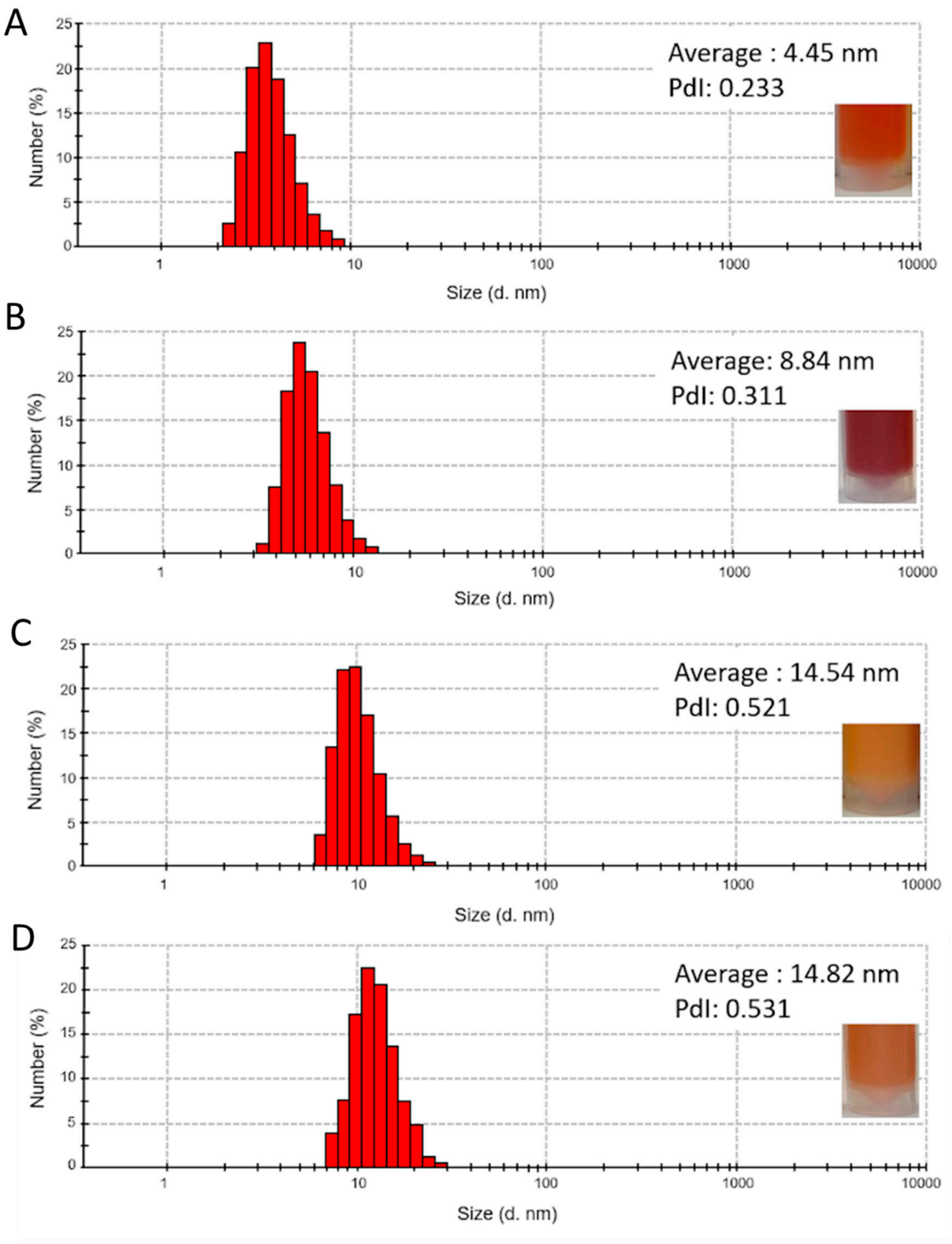

Figure S5: Size evaluation of biosynthesized nanoparticles. The size was determined using dynamic light scattering (DLS), A and B correspond to the synthesis performed with 50 mM cysteine at 14 and 30 days respectively. C and D correspond to the synthesis with 100 mM cysteine at 14 and 30 days respectively.

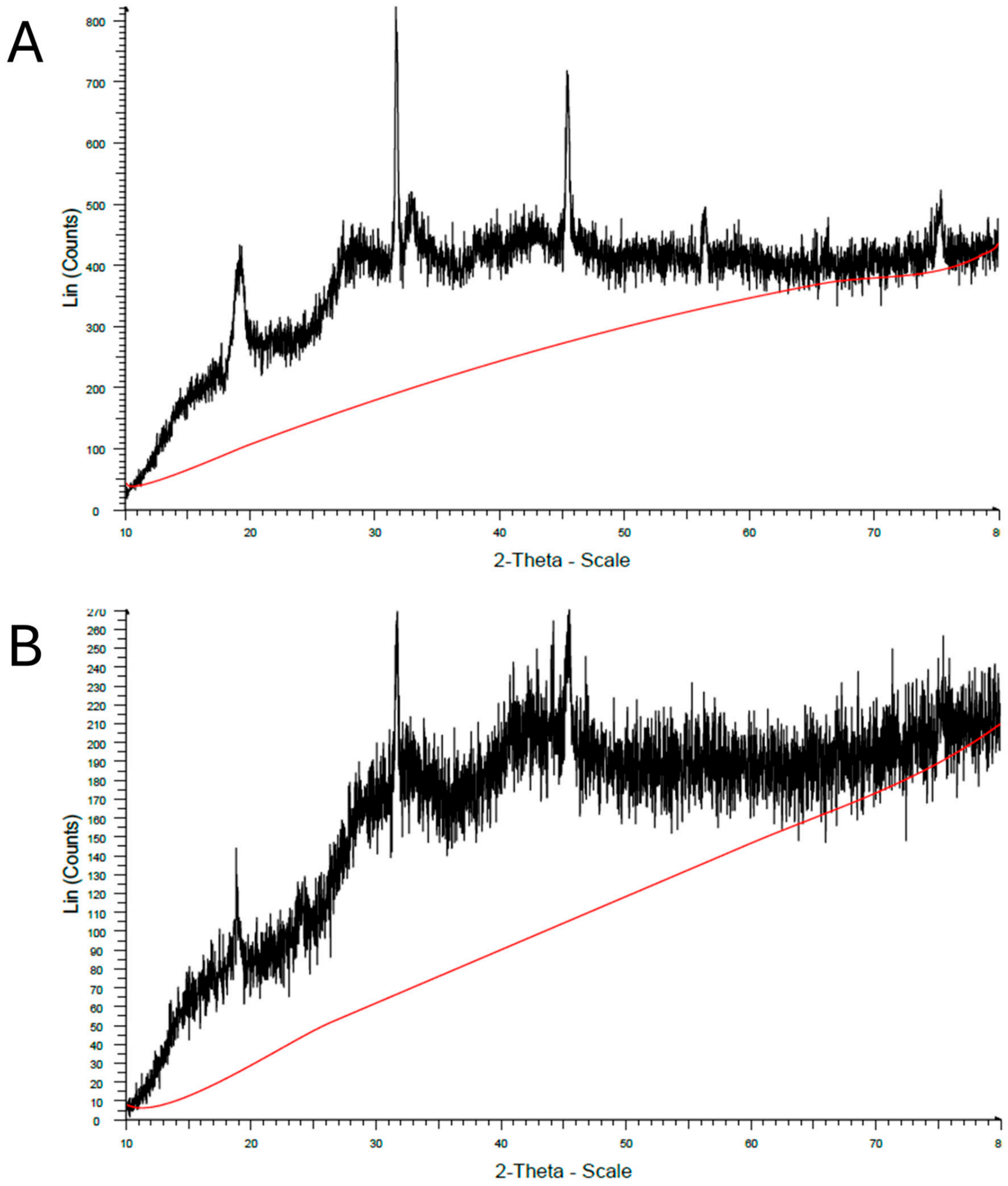

Figure S6: XRD analysis of CIS nanoparticles. (A & B) Diffraction patterns of CIS nanoparticles synthesized at 14 and 30 days of incubation, respectively. The red line represents the area of the amorphous component.

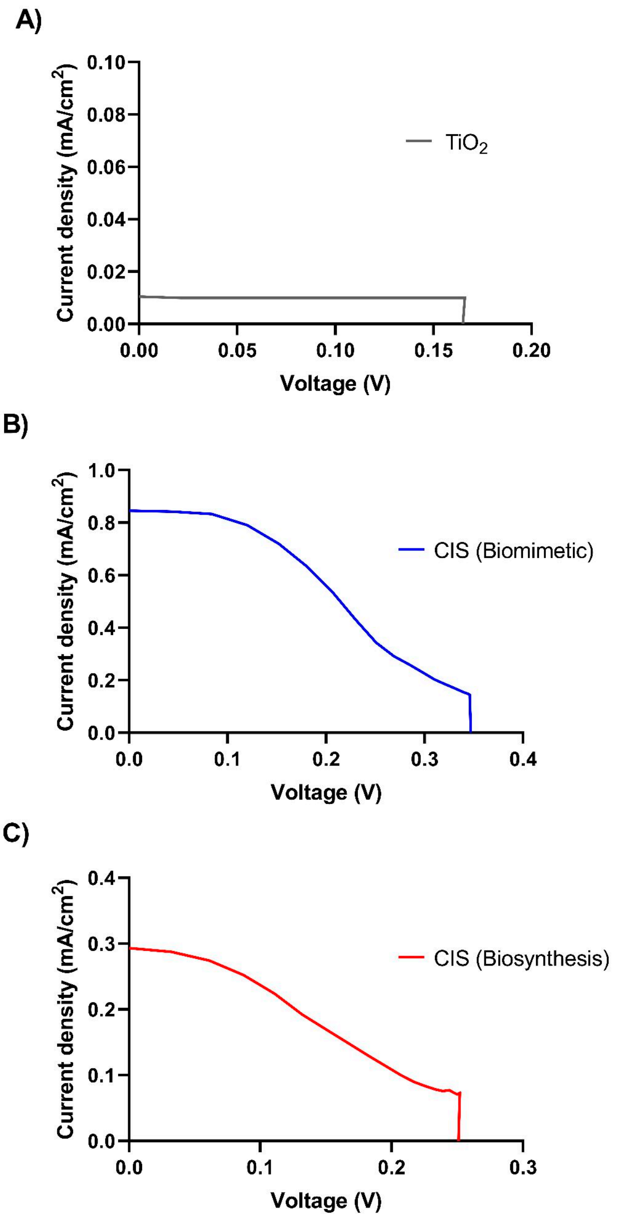

Figure S7: Current-voltage (I-V) curves of solar cells. A) TiO_2, B) CIS (biomimetic) and C) CIS (biosynthesis).

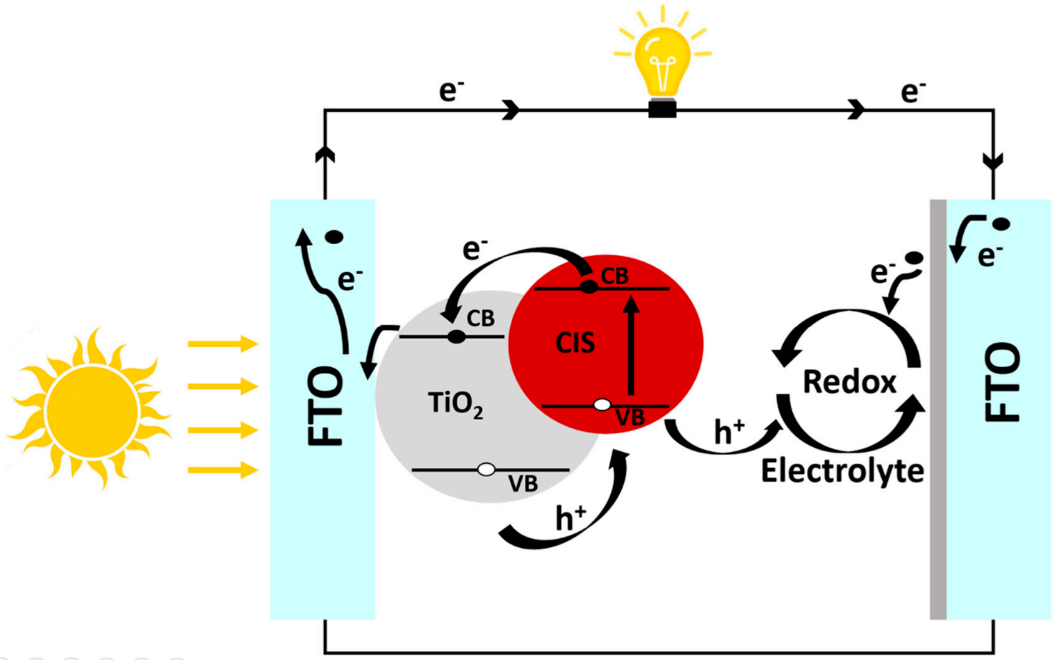

Figure S8: Schematization of the operation mechanism of quantum dot sensitized solar cells (QDSSC).
